# Physicians’ Judgments About Off-Label Medical Narcotic Use and Dose Reduction Responses: A South Korean Cross-Sectional Survey

**DOI:** 10.3390/medicina62091748

**Published:** 2026-09-10

**Authors:** Yongsoo Lee, Yang-Ki Minn, Jung Eun Kim

**Affiliations:** 1 Department of Anesthesiology and Pain Medicine, Hallym University Kangnam Sacred Heart Hospital, Seoul 07441, Republic of Korea; thisisrio@naver.com; 2 Department of Neurology, Hallym University Kangnam Sacred Heart Hospital, Seoul 07441, Republic of Korea; 3 Department of Anesthesiology and Pain Medicine, Kyung Hee University College of Medicine, Seoul 02447, Republic of Korea

**Keywords:** analgesics, opioid, attitude of health personnel, drug and narcotic control, narcotics, off-label use, Republic of Korea, practice patterns, physicians’, prescription drug misuse

## Abstract

*Background and Objectives*: Off-label medical narcotic use may be classified as misuse and abuse, but this classification need not coincide with judgments about how broadly medical narcotic use should be permitted. We examined the association and discordance between these judgments, as well as how each was related to three dose-reduction-related responses. *Materials and Methods:* We analyzed cross-sectional web-based survey data from 300 physicians specializing in neurology, psychiatry, or anesthesiology and pain medicine in South Korea. Strict classification of all off-label use as misuse and abuse and a restrictive view of how broadly medical narcotic use should be permitted were entered simultaneously into logistic regression models for prior recommendation of dose reduction or discontinuation, belief that dose reduction would be helpful, and future intention to attempt dose reduction. Models were adjusted for age, sex, practice type, and specialty, with false-discovery rate (FDR) adjustment across six primary associations. *Results:* Strict classification was selected by 42 physicians (14.0%), and a restrictive view by 107 (35.7%). The two judgments were positively associated (adjusted odds ratio [aOR], 4.99; 95% confidence interval [CI], 2.38–10.46), but 92 of 297 physicians (31.0%) gave discordant responses. After FDR adjustment, strict classification was associated with prior recommendation (aOR, 3.06; 95% CI, 1.37–6.83) and the belief that dose reduction would be helpful (aOR, 4.51; 95% CI, 1.91–10.67). A restrictive view was associated with the same belief (aOR, 3.59; 95% CI, 2.12–6.10) and future intention (aOR, 2.89; 95% CI, 1.55–5.40). *Conclusions:* These findings indicate that physicians’ broad judgments about off-label medical narcotic use and the permitted scope of medical narcotic use are related but distinct and show different patterns of association with three dose-reduction-related responses. They support the view that tapering decisions should be guided by individualized clinical assessment rather than by legal and regulatory frameworks alone.

## 1. Introduction

Physicians who prescribe medical narcotics, including opioid analgesics and controlled psychotropic medications, must balance therapeutic benefits against the risks of dependence and dose escalation while remaining alert to possible misuse and abuse [1,2]. Dependence and dose escalation, however, may occur during medically indicated treatment and are not in themselves indicators of misuse and abuse [3,4]. This distinction becomes particularly important when the appropriateness of ongoing treatment is reassessed [2,5]. An overly broad classification of medical narcotic use as misuse and abuse may unnecessarily restrict clinically appropriate treatment, whereas an overly narrow classification may delay recognition of genuinely problematic use [3,6]. These considerations have particular regulatory relevance in South Korea, where medical narcotics and illicit drugs are regulated under the same statutory framework [7].

Against this background, off-label prescribing poses a distinct classification challenge. Off-label prescribing is common in office-based practice [8], and off-label use encompasses diverse clinical circumstances, ranging from uses with limited supporting evidence to those supported by guidelines or clinical evidence but not yet reflected in the approved label [9,10,11]. Off-label status alone therefore does not establish that a particular use constitutes misuse and abuse [3,9]. A physician may consider such off-label use clinically appropriate while still believing that medical narcotic use should be restricted because of broader societal concerns. How physicians classify off-label use may thus be related to their views on how broadly medical narcotic use should be permitted, but the two judgments need not coincide.

Decisions about whether to continue treatment at the current dose, reduce the dose, or discontinue treatment provide a clinically relevant context in which to examine these judgments [1,5]. Prior recommendations, current beliefs, and future intentions capture different aspects of how physicians approach dose reduction [12]. Although previous surveys of primary care physicians examined knowledge and self-reported practices related to opioid prescribing and opioid use disorder [13,14], it remains unclear how strongly the two judgments are associated, how often they are discordant, and how each relates to physicians’ dose-reduction-related responses.

Accordingly, this study examined how physicians’ classifications of off-label medical narcotic use as misuse and abuse related to their views on how broadly medical narcotic use should be permitted. It also assessed the frequency of concordant and discordant responses. Finally, it evaluated the associations of the two judgments with three physician-reported responses: prior recommendation of dose reduction or discontinuation, the belief that dose reduction would be helpful, and future intention to attempt dose reduction.

## 2. Materials and Methods

### 2.1. Study Design, Ethics, and Analytic Overview

This study analyzed physician responses from a cross-sectional survey conducted in South Korea on perceptions and experiences related to medical narcotics. A patient–physician comparison using data from the same survey was reported previously [15], whereas the present study focused exclusively on physician responses. The analyses examined the association between a strict classification of off-label use as misuse and abuse and a restrictive view of how broadly medical narcotic use should be permitted. They also described concordant and discordant response patterns based on cross-classification of the two judgments. Primary multivariable models estimated the mutually adjusted associations of the two focal variables with each of the three physician-reported dose-reduction-related responses. Alternative and sensitivity models, as well as exploratory and descriptive analyses, are detailed in Section 2.5.

The study was reported in accordance with the STROBE statement and the CHERRIES checklist; completed checklists are provided in Supplementary Appendixes S1 and S2. The study was conducted in accordance with the Declaration of Helsinki, and the protocol was approved by the Institutional Review Board of Hallym University Kangnam Sacred Heart Hospital (IRB No. 2020-09-001; 14 October 2020).

### 2.2. Participants and Survey Administration

Board-certified physicians specializing in neurology, psychiatry, or anesthesiology and pain medicine were identified through the Korean Medical Association email database and invited to participate by email. These specialties were selected a priori because a preliminary review of Narcotics Information Management System (NIMS) prescription records identified them among the specialties with the highest prescription frequencies for opioid analgesics and controlled psychotropic medications [15]. Physicians were eligible only if they reported prior experience prescribing medical narcotics in response to a presurvey screening question.

A professional survey agency administered the web-based survey. Each email invitation contained an individualized, secure survey link, and the platform permitted only one submission per invitee. Before beginning the survey, eligible physicians were informed of the study purpose, estimated completion time (5–10 min), voluntary nature of participation, and data-handling procedures; electronic informed consent was then obtained. The survey remained open for 8 weeks, from 15 November 2021 to 10 January 2022, with three reminder emails sent at 2-week intervals. No incentives were offered. Responses were de-identified and assigned unique study identifiers before analysis.

The original survey targeted 300 physician respondents on the basis of a previously reported sample-size calculation [15]. To account for anticipated nonresponse, 800 physicians were invited. Of these, 309 completed the survey (38.6% of invitees). Nine responses were excluded because the reported age and years of clinical experience were internally inconsistent, yielding a final analytic sample of 300 physicians.

### 2.3. Survey Instrument and Variables

The Korean-language physician questionnaire was developed through a literature review and content-validity assessment by a six-member expert panel, yielding a scale-level content validity index based on the average method (S-CVI/Ave) of 0.87. The questionnaire was further refined through cognitive interviews with 10 physicians [15]. Because it was not designed to measure latent constructs, no formal factor-analytic evaluation of its dimensional structure was performed. The English wording presented in this manuscript was lightly edited for readability while preserving the substantive meaning of the original Korean items.

The original Korean questionnaire used a single term encompassing both misuse and abuse. Accordingly, “misuse and abuse” is used throughout this manuscript as an umbrella term; the questionnaire was not designed to distinguish misuse from abuse as separate legal, psychiatric, or diagnostic categories.

Q1 and Q2 served as the two focal variables, representing physicians’ classification of off-label use as misuse and abuse and their views on how broadly medical narcotic use should be permitted, respectively. Q3–Q5 assessed three physician-reported dose-reduction-related responses: prior recommendation of dose reduction or discontinuation, belief that dose reduction would be helpful, and future intention to attempt dose reduction. Because these items represented temporally and conceptually different responses—a reported prior recommendation, a current belief, and a stated future intention—they were analyzed separately rather than combined into a composite measure. They were interpreted as physician-reported responses rather than verified prescribing behavior or tapering practice. Q6–Q10 assessed demographic and professional characteristics, and Q11–Q19 comprised nine individual contextual items.

For descriptive organization, Q11–Q19 were divided into three item groups: narcotics knowledge and awareness (Q11–Q13), regulatory and control-system-related awareness (Q14–Q17), and perceived patient misuse and related use patterns (Q18–Q19). These groupings were not conceived as internally coherent scales; previously reported internal consistency estimates likewise did not support treating them as such [15]. Accordingly, no composite scores were constructed, and each item was examined separately in exploratory item-level models. Descriptive response distributions for Q11–Q19 were reported previously [15], whereas the present study evaluated their adjusted item-level associations.

For exploratory system-context analyses, Q16 and Q17 were selected because they assessed awareness of the NIMS Data Service and willingness to try it, respectively. The full item wording and response options are provided in Supplementary Appendix S3.

### 2.4. Analytic Variable Definitions

For analysis, responses to Q1 and Q2 were grouped into binary categories. A strict misuse-and-abuse classification was defined as Q1 option 1, which classified all use outside the label approved by the Ministry of Food and Drug Safety (MFDS) as misuse and abuse. Options 2–4 were combined into the non-strict category. A restrictive view was defined as Q2 option 1, which indicated that medical narcotic use, including off-label use, should be restricted because misuse and abuse are serious societal concerns. Options 2 and 3 were combined into the non-restrictive category. The non-restrictive category pooled respondents who believed that medical narcotic use, including off-label use, should be permitted at the physician’s discretion and those who believed that it should be permitted when there is a clear medical need; it should therefore not be interpreted as endorsement of unrestricted use. The “Unsure” response was not assigned to either binary view category.

For the combined-pattern analysis, the binary Q1 and Q2 categories were cross-classified, yielding four patterns: strict–restrictive, strict–non-restrictive, non-strict–restrictive, and non-strict–non-restrictive. The first descriptor refers to misuse-and-abuse classification, and the second refers to the view on how broadly medical narcotic use should be permitted. These patterns were descriptive analytic groupings rather than distinct respondent types. Because the non-strict and non-restrictive categories each combined multiple response options, neither should be assumed to represent a homogeneous set of views.

Q3–Q5 and Q11–Q19 were analyzed as binary variables (yes versus no). Age was analyzed as a continuous variable, whereas sex, practice type, and specialty were analyzed as categorical variables. Years of clinical experience were used only for demographic consistency checks and were not included in the regression models to avoid redundancy with age.

### 2.5. Statistical Analysis

Continuous variables were summarized as medians with interquartile ranges (IQR), and categorical variables as counts and percentages. There were no missing data for the variables included in the analyses. Analyses requiring the binary restrictive-view variable excluded the three respondents who selected “Unsure,” leaving 297 physicians; analyses that did not require this variable retained all 300 physicians.

The two focal variables were cross-tabulated, and their adjusted association was estimated using multivariable logistic regression with a restrictive view as the outcome and strict misuse-and-abuse classification as the predictor. This model direction was used for estimation only and did not imply temporal or causal ordering. All regression models included age, sex, practice type, and specialty as adjustment covariates.

Separate multivariable logistic regression models were fitted for Q3, Q4, and Q5. The primary models included both focal variables simultaneously. Alternative models included one focal variable at a time. Expanded sensitivity models retained all primary-model terms and added all nine contextual items (Q11–Q19) simultaneously. Exploratory system-context models added Q16 and Q17 simultaneously to the primary-model terms.

For the exploratory item-level analyses, each Q11–Q19 item was entered separately as the predictor in models for each of the two focal outcomes: strict misuse-and-abuse classification and a restrictive view. This yielded 18 item-level associations, nine for each focal outcome.

For each of the four combined response patterns, adjusted predicted probabilities of affirmative responses to Q3–Q5 were obtained from the primary models by marginal standardization [16]. The two focal variables were set to each of the four response combinations, and the resulting predictions were averaged over the observed covariate distribution. The corresponding 95% confidence intervals were derived using the delta method. No formal between-pattern comparisons were performed.

Adjusted odds ratios (aORs) with 95% confidence intervals (CIs) were reported. The Benjamini–Hochberg procedure was used to control the false-discovery rate (FDR) separately within two families: the six primary associations of the two focal variables with Q3–Q5 and the 18 exploratory item-level associations of Q11–Q19 with the two focal outcomes. Within these families, inference was based on FDR-adjusted *p*-values. All other inferential analyses used two-sided nominal *p*-values, with *p* < 0.05 regarded as nominal evidence of association; 95% CIs were not adjusted for multiplicity.

All analyses were conducted using Python version 3.13.5; logistic regression models were fitted with statsmodels version 0.14.6.

## 3. Results

### 3.1. Physician Characteristics

The final analytic sample comprised 300 physicians. The median age was 49 years (IQR, 43–55), and 250 physicians (83.3%) were male. Private practitioners constituted the largest practice group (43.7%), followed by university hospital physicians (41.3%) and employed physicians (15.0%). Psychiatry was the most common specialty (64.3%), followed by neurology (27.3%) and anesthesiology and pain medicine (8.3%) (Table 1).

### 3.2. Distribution of Survey Responses

Table 2 presents the distributions of the two focal variables and the three physician-reported dose-reduction-related responses. A strict misuse-and-abuse classification of off-label use was selected by 42 physicians (14.0%), whereas the remaining 258 physicians (86.0%) selected one of the three non-strict responses. A restrictive view was selected by 107 physicians (35.7%); 190 physicians (63.3%) selected one of the two non-restrictive responses; and 3 (1.0%) selected “Unsure.”

Among the three dose-reduction-related responses, 153 physicians (51.0%) reported having previously recommended dose reduction or discontinuation, 129 (43.0%) believed that dose reduction would be helpful, and 212 (70.7%) reported an intention to attempt dose reduction.

### 3.3. Association and Discordance Between Strict Misuse-and-Abuse Classification and a Restrictive View

Among the 297 physicians who selected a Q2 response other than “Unsure,” strict misuse-and-abuse classification and a restrictive view were positively associated but not fully concordant. In the cross-tabulation, 27 physicians had both a strict classification and a restrictive view, whereas 178 had neither. Discordant responses were observed in 92 physicians (31.0%): 12 had a strict classification with a non-restrictive view, and 80 had a non-strict classification with a restrictive view (Supplementary Table S1).

After adjustment for age, sex, practice type, and specialty, physicians with a strict misuse-and-abuse classification had higher odds of holding a restrictive view (aOR, 4.99; 95% CI, 2.38–10.46; nominal *p* < 0.001). Thus, the two judgments were positively associated, but discordance was observed in nearly one-third of physicians.

### 3.4. Primary Associations with Physician-Reported Dose-Reduction-Related Responses

In the primary mutually adjusted models, both focal variables were entered simultaneously, with adjustment for age, sex, practice type, and specialty (Table 3).

Strict misuse-and-abuse classification was associated with prior recommendation of dose reduction or discontinuation (Q3; aOR, 3.06; 95% CI, 1.37–6.83; FDR-adjusted *p* = 0.010) and belief that dose reduction would be helpful (Q4; aOR, 4.51; 95% CI, 1.91–10.67; FDR-adjusted *p* = 0.002). Although the point estimate for its association with future intention to attempt dose reduction was above 1, the association did not meet the FDR-adjusted significance criterion (Q5; aOR, 2.96; 95% CI, 0.97–9.02; FDR-adjusted *p* = 0.067).

A restrictive view was associated with belief that dose reduction would be helpful (Q4; aOR, 3.59; 95% CI, 2.12–6.10; FDR-adjusted *p* < 0.001) and future intention to attempt dose reduction (Q5; aOR, 2.89; 95% CI, 1.55–5.40; FDR-adjusted *p* = 0.002). Although the point estimate for its association with prior recommendation of dose reduction or discontinuation was above 1, the association did not meet the FDR-adjusted significance criterion (Q3; aOR, 1.41; 95% CI, 0.84–2.35; FDR-adjusted *p* = 0.193).

Belief that dose reduction would be helpful (Q4) was associated with both focal variables after FDR adjustment. In contrast, prior recommendation of dose reduction or discontinuation (Q3) was associated only with strict misuse-and-abuse classification, whereas future intention to attempt dose reduction (Q5) was associated only with a restrictive view.

### 3.5. Alternative, Sensitivity, Combined-Pattern, and Exploratory Analyses

Primary inference was based on the mutually adjusted models presented in Table 3. In alternative models that included one focal variable at a time and adjusted for age, sex, practice type, and specialty, both focal variables were nominally associated with all three dose-reduction-related responses (Supplementary Table S2, Panel A). Expanded sensitivity models that additionally included all nine contextual items (Q11–Q19) yielded estimates in the same direction as the primary models (Supplementary Table S2, Panel B). In these models, strict misuse-and-abuse classification was nominally associated with all three responses, whereas a restrictive view was nominally associated with belief that dose reduction would be helpful and future intention to attempt dose reduction; its association with prior recommendation of dose reduction or discontinuation was in the same direction but did not reach nominal statistical significance.

Adjusted predicted probabilities summarized the primary mutually adjusted results on the probability scale across the four combined response patterns (Supplementary Table S3). For the belief that dose reduction would be helpful, the predicted probability was lowest in the non-strict–non-restrictive pattern, at 28.0% (95% CI, 22.0–34.9); intermediate in the non-strict–restrictive and strict–non-restrictive patterns, at 57.9% and 63.2%, respectively; and highest in the strict–restrictive pattern, at 85.9% (95% CI, 72.4–93.4). For prior recommendation of dose reduction or discontinuation, the predicted probabilities were 70.3–76.7% in the two strict patterns and 44.7–52.8% in the two non-strict patterns. For future intention to attempt dose reduction, the predicted probability was 61.3% in the non-strict–non-restrictive pattern, 81.4–81.8% in the two discordant patterns, and 92.7% in the strict–restrictive pattern. These descriptive estimates were consistent with the patterns of association observed in the primary mutually adjusted models. No formal between-pattern comparisons were performed.

In exploratory system-context models, awareness of the NIMS Data Service was nominally associated with belief that dose reduction would be helpful (aOR, 2.71; 95% CI, 1.54–4.76; nominal *p* = 0.001) and future intention to attempt dose reduction (aOR, 2.49; 95% CI, 1.33–4.69; nominal *p* = 0.005), but did not show nominal evidence of association with prior recommendation of dose reduction or discontinuation (aOR, 1.27; 95% CI, 0.75–2.14; nominal *p* = 0.378). Willingness to try the service did not show nominal evidence of association with any of the three responses (Supplementary Figure S1).

In exploratory item-level analyses of Q11–Q19, self-reported ability to distinguish medical narcotics from illicit drugs was inversely associated with both focal outcomes, whereas awareness that physicians may decline a clinically inappropriate medical narcotic prescription request was positively associated with a restrictive view. These were the only item-level associations that remained statistically significant after FDR adjustment (Supplementary Table S4).

## 4. Discussion

Strict misuse-and-abuse classification of off-label medical narcotic use and a restrictive view of how broadly such use should be permitted were positively associated but not interchangeable. In the cross-tabulation, physicians who endorsed strict classification were more likely to hold a restrictive view, yet nearly one third of respondents showed discordant patterns, combining non-strict classification with a restrictive view or strict classification with a non-restrictive view. These findings indicate that the two judgments are related but distinct and capture overlapping yet different dimensions of how physicians conceptualize appropriate medical narcotic use. Strict classification was associated with prior recommendation and perceived helpfulness of dose reduction, whereas a restrictive view was associated with perceived helpfulness and future intention to attempt dose reduction, suggesting that definitional judgments and broader concerns about permissibility may each be related to physicians’ reported dose-reduction-related responses.

The two questions differ in kind, which helps explain why physicians’ responses diverged. The first question is primarily definitional: it asks whether all off-label medical narcotic use should be classified as misuse and abuse. The second concerns scope: it asks how broadly medical narcotic use, including off-label use, should be permitted in the context of societal concerns about misuse and abuse. The response options for the first question distinguished off-label use recognized in clinical guidelines or textbooks from off-label use based solely on an individual physician’s prescribing decision. A physician may therefore reserve the classification of misuse and abuse for a narrower set of circumstances, recognizing that some off-label use can be clinically supported, while still holding a restrictive view of how broadly medical narcotic use should be permitted in light of broader societal concerns and regulatory scrutiny surrounding controlled-medication prescribing. This combination accounted for 80 of the 92 discordant responses. The less common pattern ran in the opposite direction, with 12 physicians classifying all off-label use as misuse and abuse while believing that medical narcotic use should remain permitted at the physician’s discretion or when there is a clear medical need; this smaller group further illustrates that strict classification did not necessarily coincide with a restrictive view of permitted use. Adjusted predicted probabilities for the four combined response patterns were consistent with the patterns observed in the primary mutually adjusted models (Supplementary Table S3); these pattern-level estimates are descriptive, and no formal between-pattern comparisons were performed. Taken together, these discordant patterns need not indicate inconsistency; from a cognitive perspective, the same considerations—approved labeling, clinical evidence, medical necessity, and societal risk—may carry different weight depending on whether physicians are classifying off-label use as misuse and abuse or judging how broadly medical narcotic use should be permitted. Because the survey did not directly assess the cognitive processes underlying these judgments, this interpretation should not be taken as evidence of a specific psychological mechanism.

An earlier report from the same survey compared patients with physicians and identified between-group differences in perceptions and awareness concerning medical narcotics and the narcotics control system [15], whereas the present analysis extends that work by examining variation within the physician sample, including discordance between the two focal judgments and the mutually adjusted association of each judgment with dose-reduction-related responses. A survey of 1007 primary care physicians in the United States likewise identified important gaps in opioid-related knowledge and self-reported practice [13] but did not examine whether physicians distinguished what should be classified as misuse and abuse from how broadly medical narcotic use should be permitted. The broader literature provides a conceptual basis for making this distinction. An ACTTION systematic review found that definitions of prescription-medication misuse, abuse, and related events varied according to the purpose of the classification system and were frequently inconsistent or overlapping [3,17]. Qualitative research on off-label prescribing similarly reported that physicians did not necessarily regard label status alone as a reliable measure of clinical appropriateness [18]. Against this background, the low prevalence of strict classification in the present sample and its incomplete correspondence with physicians’ views on how broadly medical narcotic use should be permitted are consistent with the conceptual distinctions described in the previous literature [3,17,18].

These findings have practical implications for physician education, clinical guidance, and the regulation of medical narcotics. Educational programs should clearly distinguish the classification of off-label use as misuse and abuse from judgments about how broadly medical narcotic use should be permitted, and should further distinguish both from individualized, patient-centered clinical judgment about whether treatment should be continued, reduced, or discontinued. They should also emphasize that off-label status alone does not establish misuse or abuse and that clinically appropriate off-label use can coexist with legitimate concerns about societal risk [3,10,18]. Clinical guidance and tapering decision aids should likewise distinguish broad classificatory and regulatory judgments from individualized, patient-centered clinical decisions. In South Korea, where medical narcotics are regulated under the Narcotics Control Act and prescription information is managed through the NIMS, regulatory and data systems should avoid treating off-label status as sufficient evidence of misuse or abuse [7,19]. Instead, they should accommodate clinically justified off-label use while supporting the identification of problematic prescribing or use patterns.

The exploratory item-level analyses identified several knowledge- and system-related correlates, although only three of the 18 item-level associations met the FDR-adjusted criterion. Self-reported ability to distinguish medical narcotics from illicit drugs was inversely associated with both strict misuse-and-abuse classification and a restrictive view, whereas awareness that physicians may decline a clinically inappropriate medical narcotic prescription request was positively associated with a restrictive view. One possible interpretation is that physicians who reported being unable to distinguish medical narcotics from illicit drugs may have expressed more precautionary judgments, classifying all off-label use as misuse and abuse and endorsing a restrictive view of how broadly medical narcotic use should be permitted. Because this item assessed self-perceived rather than objectively tested knowledge, this interpretation remains tentative. In separate exploratory system-context analyses, awareness of the NIMS Data Service showed nominal associations with belief that dose reduction would be helpful and with future intention to attempt dose reduction, but not with prior recommendation, whereas willingness to try the service was not associated with any of the three responses. A small qualitative study of Korean physicians and pharmacists reported that participants used NIMS mainly for data reporting and identified a need for more clinically usable feedback and medication-check functions [20]. Taken together, these findings suggest that awareness of a system resource is not equivalent to its integration into clinical decision-making [21].

The central clinical implication of these findings is that three related but non-equivalent questions should remain distinct: whether off-label use should be classified as misuse and abuse, how broadly medical narcotic use should be permitted, and whether treatment should be continued, reduced, or discontinued for an individual patient. The first two judgments may inform clinical assessment but should not by themselves determine the third [22]. Accordingly, off-label status or a general classification of such use as misuse and abuse should not, by itself, be treated as sufficient grounds for dose reduction or discontinuation. Contemporary guidance for opioid treatment and for medicines associated with dependence or withdrawal similarly emphasizes individualized assessment rather than rigid application of categorical rules [1,2,5,23,24].

Key strengths of this study were the separate measurement and simultaneous modeling of the two focal judgments and the retention of prior recommendation, current belief, and future intention as distinct responses. The analytic approach also addressed multiplicity through FDR adjustment within clearly defined families of primary and item-level analyses. Several limitations should be acknowledged. First, the cross-sectional email survey precludes causal or directional inference and is susceptible to selection and nonresponse bias. Of the 800 invited physicians, 309 completed the survey (38.6% of invitees), and nonrespondents could not be characterized. Second, all measures relied on self-report and were therefore susceptible to recall and social-desirability bias. In particular, the knowledge and awareness items assessed physicians’ self-perceptions rather than objectively tested knowledge or observed behavior, and the corresponding exploratory associations may reflect unmeasured differences in training or clinical experience. The survey also did not capture the specific medical narcotic, indication, dose, treatment duration, or clinical rationale; the dose-reduction-related items therefore represented reported recommendations, beliefs, and intentions rather than verified treatment changes or patient outcomes. Third, the focal judgments and contextual factors were assessed with single items, and dichotomization reduced distinctions among the original response levels within the non-strict and non-restrictive categories. The Korean questionnaire also used one umbrella term for misuse and abuse without distinguishing among legal, psychiatric, diagnostic, and behavioral interpretations of these concepts. Because misuse and abuse have distinct meanings in clinical pharmacology and related disciplines, the use of a single umbrella term may also have influenced how respondents interpreted and answered the relevant items. Fourth, the combined response patterns were descriptive groupings rather than validated physician types, and the strict–non-restrictive subgroup included only 12 physicians. Fifth, findings from the exploratory analyses, particularly those based on nominal *p* values, should be considered hypothesis-generating. Finally, the sample was limited to three specialties in a single regulatory setting and was predominantly composed of psychiatrists. Because prescribing contexts may differ across specialties, the observed response distributions may disproportionately reflect perspectives associated with controlled psychotropic medication prescribing; the findings should therefore be generalized cautiously, particularly with respect to opioid-focused pain practice and specialties not represented in the sample.

## 5. Conclusions

In this survey of South Korean physicians, strict misuse-and-abuse classification of off-label medical narcotic use and a restrictive view of how broadly such use should be permitted were positively associated but distinct. The two judgments also showed different patterns of association with the three dose-reduction-related responses. These findings support the view that tapering decisions should be guided by individualized clinical assessment rather than by general legal and regulatory frameworks alone. They also highlight the need for clearer operational definitions and transparent clinical criteria that distinguish these two judgments from patient-level decisions about whether and how to reduce or discontinue medical narcotic treatment.

## Figures and Tables

**Table 1:** Characteristics of participating physicians.

Characteristic	Overall (*n* = 300)
Age, years	49 (43–55)
Sex	
Male	250 (83.3)
Female	50 (16.7)
Practice type	
Private practitioner	131 (43.7)
University hospital physician	124 (41.3)
Employed physician	45 (15.0)
Medical specialty	
Psychiatry	193 (64.3)
Neurology	82 (27.3)
Anesthesiology and pain medicine	25 (8.3)

Values are presented as median (IQR) or *n* (%). IQR, interquartile range.

**Table 2:** Distributions of the two focal variables and physician-reported dose-reduction-related responses.

(A) Misuse-and-abuse classification and view on how broadly medical narcotic use should be permitted.				
**Survey Domain**	**Question**	**Response Category**	**Binary Analytic Category**	***n*** **(%)**
Misuse-and-abuse classification of off-label use	Q1	All off-label use classified as misuse and abuse	Strict	42 (14.0)
		Guideline-recognized off-label use not classified as misuse and abuse	Non-strict	139 (46.3)
		Textbook-recognized off-label use not classified as misuse and abuse	Non-strict	80 (26.7)
		Physician-prescribed off-label use not classified as misuse and abuse	Non-strict	39 (13.0)
View on how broadly medical narcotic use, including off-label use, should be permitted	Q2	Should be restricted because of serious societal concerns about misuse and abuse	Restrictive	107 (35.7)
		Should be permitted at the physician’s discretion	Non-restrictive	31 (10.3)
		Should be permitted when there is a clear medical need	Non-restrictive	159 (53.0)
		Unsure	Not assigned	3 (1.0)
(B) Physician-reported dose-reduction-related responses				
**Survey Domain**	**Question**	**Label**		**Yes,** ***n*** **(%)**
Dose reduction-related responses	Q3	Prior recommendation of dose reduction or discontinuation		153 (51.0)
	Q4	Belief that dose reduction would be helpful		129 (43.0)
	Q5	Future intention to attempt dose reduction		212 (70.7)

Values are presented as *n* (%); percentages were calculated using all 300 physicians as the denominator. In Panel B, values represent affirmative responses. The Q2 “Unsure” response was not assigned to either binary analytic category and was excluded from analyses requiring the binary restrictive-view variable. Response-category wording has been shortened for readability; the full item wording and response options are provided in Supplementary Appendix S3.

**Table 3:** Primary mutually adjusted associations of strict misuse-and-abuse classification and a restrictive view with physician-reported dose-reduction-related responses.

Physician-Reported Response	Strict Misuse-and-Abuse Classification		Restrictive View	
	aOR (95% CI)	FDR-Adjusted *p*-Value	aOR (95% CI)	FDR-Adjusted *p*-Value
Prior recommendation of dose reduction or discontinuation (Q3)	3.06 (1.37–6.83)	0.010	1.41 (0.84–2.35)	0.193
Belief that dose reduction would be helpful (Q4)	4.51 (1.91–10.67)	0.002	3.59 (2.12–6.10)	<0.001
Future intention to attempt dose reduction (Q5)	2.96 (0.97–9.02)	0.067	2.89 (1.55–5.40)	0.002

Effect estimates are presented as adjusted odds ratios (aORs) with 95% confidence intervals (CIs). Analyses included the 297 physicians who selected a Q2 response other than “Unsure.” For each physician-reported response, a separate logistic regression model included both focal variables and was adjusted for age, sex, practice type, and specialty. Strict misuse-and-abuse classification was defined as Q1 response option 1 versus response options 2–4; a restrictive view was defined as Q2 response option 1 versus response options 2 and 3. FDR-adjusted *p*-values were calculated using the Benjamini–Hochberg procedure across the six associations shown; the 95% CIs were nominal and were not adjusted for multiple comparisons. FDR, false-discovery rate.

## Data Availability

The datasets used and/or analyzed in the current study are available from the corresponding author on reasonable request.

## Supplementary Materials

The following supporting information can be downloaded at https://www.mdpi.com/article/10.3390/medicina62091748/s1. Table S1: Cross-tabulation of strict misuse-and-abuse classification and a restrictive view; Table S2: Associations of strict misuse-and-abuse classification and a restrictive view with physician-reported dose-reduction-related responses in alternative and expanded sensitivity models; Table S3: Adjusted predicted probabilities of physician-reported dose-reduction-related responses across the four combined response patterns; Table S4: Exploratory associations of individual contextual items (Q11–Q19) with strict misuse-and-abuse classification and a restrictive view; Figure S1: Exploratory associations of system-context items with physician-reported dose-reduction-related responses. Appendix S1: STROBE checklist; Appendix S2: CHERRIES checklist; Appendix S3: Wording and response options of survey items used in the present study.

## Abbreviations

The following abbreviations are used in this manuscript:

MFDS	Ministry of Food and Drug Safety
NIMS	Narcotics Information Management System

## References

[1] Dowell D., Ragan K.R., Jones C.M., Baldwin G.T., Chou R. (2022). CDC clinical practice guideline for prescribing opioids for pain—United States, 2022. MMWR. Recomm. Rep..

[2] Carville S., Lally M., Stannard C., Trewern L. (2022). Medicines associated with dependence or withdrawal symptoms: Summary of NICE guideline. BMJ.

[3] Smith S.M., Dart R.C., Katz N.P., Paillard F., Adams E.H., Comer S.D., Degroot A., Edwards R.R., Haddox J.D., Jaffe J.H. (2013). Classification and definition of misuse, abuse, and related events in clinical trials: ACTTION systematic review and recommendations. PAIN.

[4] Volkow N.D., McLellan A.T. (2016). Opioid abuse in chronic pain—Misconceptions and mitigation strategies. N. Engl. J. Med..

[5] Dowell D., Compton W.M., Giroir B.P. (2019). Patient-centered reduction or discontinuation of long-term opioid analgesics: The HHS guide for clinicians. JAMA.

[6] World Health Organization (2025). WHO Guideline on Balanced National Controlled Medicines Policies to Ensure Medical Access and Safety.

[7] Korea Legislation Research Institute (2016). Narcotics Control Act, Article 2. Statutes of the Republic of Korea.

[8] Radley D.C., Finkelstein S.N., Stafford R.S. (2006). Off-label Prescribing Among Office-Based Physicians. Arch. Intern. Med..

[9] Gazarian M., Kelly M., McPhee J.R., Graudins L.V., Ward R.L., Campbell T.J. (2006). Off-label use of medicines: Consensus recommendations for evaluating appropriateness. Med. J. Aust..

[10] Gazarian M., Horton D.B., Carleton B., Kinlaw A.C., Bushnell G.A., Czaja A.S., Durrieu G., Gorman E.F., Titievsky L., Zito J. (2023). Optimizing therapeutic decision-making for off-label medicines use: A scoping review and consensus recommendations for improving practice and research. Pharmacoepidemiol. Drug Saf..

[11] Wittich C.M., Burkle C.M., Lanier W.L. (2012). Ten common questions (and their answers) about off-label drug use. Mayo Clin. Proc..

[12] Godin G., Bélanger-Gravel A., Eccles M., Grimshaw J. (2008). Healthcare professionals’ intentions and behaviours: A systematic review of studies based on social cognitive theories. Implement. Sci..

[13] Ragan-Burnett K.R., Curtis C.R., Schmit K.M., Mikosz C.A., Schieber L.Z., Guy G.P., Haegerich T.M. (2024). Physicians’ Self-Reported Knowledge and Behaviors Related to Prescribing Opioids for Chronic Pain and Diagnosing Opioid Use Disorder, DocStyles, 2020. AJPM Focus.

[14] Hwang C.S., Turner L.W., Kruszewski S.P., Kolodny A., Alexander G.C. (2016). Primary care physicians’ knowledge and attitudes regarding prescription opioid abuse and diversion. Clin. J. Pain.

[15] Lee Y., Chun E.H., Minn Y.-K., Ahn S.H., Kim J.H., Kang H.Y., Lee H.S., Kim J.E. (2025). Perceptual Discrepancies of Opioid Analgesics and Psychotropic Drugs: A Cross-Sectional Study of Korean Patients and Physicians. J. Clin. Med..

[16] Muller C.J., MacLehose R.F. (2014). Estimating predicted probabilities from logistic regression: Different methods correspond to different target populations. Int. J. Epidemiol..

[17] Barrett S.P., Meisner J.R., Stewart S.H. (2008). What constitutes prescription drug misuse? Problems and pitfalls of current conceptualizations. Curr. Drug Abus. Rev..

[18] Ghinea N., Kerridge I., Little M., Lipworth W. (2017). Challenges to the validity of using medicine labels to categorize clinical behavior: An empirical and normative critique of “off-label” prescribing. J. Eval. Clin. Pract..

[19] Kim S.-Y., Cho N.-W., Yoo M.-S., Han S.-Y., Oh J.-W. (2023). Narcotics information management system in South Korea: System development and innovation. BMC Health Serv. Res..

[20] Kim J., Shin Y.-j. (2023). Qualitative Study on the Narcotics Information Management System (NIMS) Experience of Doctors and Pharmacists Using Narcotic Analgesics. Korean J. Clin. Pharm..

[21] Leichtling G.J., Irvine J.M., Hildebran C., Cohen D.J., Hallvik S.E., Deyo R.A. (2017). Clinicians’ Use of Prescription Drug Monitoring Programs in Clinical Practice and Decision-Making. Pain Med..

[22] Bhattacharya D., Whiteside H., Tang E., Kantilal K., Loke Y., Atkins B., Hill C. (2022). A review of trial and real-world data applying elements of a realist approach to identify behavioural mechanisms supporting practitioners to taper opioids. Br. J. Clin. Pharmacol..

[23] Langford A.V., Lin C.C., Bero L., Blyth F.M., Doctor J., Holliday S., Jeon Y.H., Moullin J., Murnion B., Nielsen S. (2023). Clinical practice guideline for deprescribing opioid analgesics: Summary of recommendations. Med. J. Aust..

[24] Kim M., Park S.K., Kim W.M., Kim E., Kim H., Park J.-M., Choi S.-S., Choi E.J. (2024). Updated guidelines for prescribing opioids to treat patients with chronic non-cancer pain in Korea: Developed by committee on hospice and palliative care of the Korean Pain Society. Korean J. Pain.

