# Supplementary material for: Physicians’ Judgments About Off-Label Medical Narcotic Use and Dose Reduction Responses: A South Korean Cross-Sectional Survey
Source: Medicina (Kaunas). 2026 Sep 10;62(9):1748. doi: 10.3390/medicina62091748 (PMC13608935; doi:10.3390/medicina62091748)
Supplement: Supplementary file 1 [file medicina-62-01748-s001.zip › Supplementary Materials.pdf]

Supplementary Materials

Supplementary Table S1. Cross-tabulation of strict misuse-and-abuse classification and a restrictive view.

Misuse-and-abuse classification	View on how broadly medical narcotic use should be permitted		Total
	Restrictive	Non-restrictive	
Strict	27 (69.2)	12 (30.8)	39
Non-strict	80 (31.0)	178 (69.0)	258
Total	107	190	297

Values in the 2 × 2 body are presented as *n* (row %); row and column totals are presented as *n*. Strict misuse-and-abuse classification was defined as selection of Q1 response option 1, whereas response options 2–4 were categorized as non-strict. A restrictive view was defined as selection of Q2 response option 1, whereas response options 2 and 3 were categorized as non-restrictive. The Q2 “Unsure” response was not assigned to either binary view category and was excluded from this cross-tabulation.

Supplementary Table S2. Associations of strict misuse-and-abuse classification and a restrictive view with physician-reported dose reduction-related responses in alternative and expanded sensitivity models.

Panel A. Alternative models including one focal variable at a time, adjusted for age, sex, practice type, and specialty

Response	Strict misuse-and-abuse classification, aOR (95% CI)	Nominal *p*-value	Restrictive view, aOR (95% CI)	Nominal *p*-value
Q3	3.38 (1.59–7.17)	0.002	1.69 (1.03–2.76)	0.037
Q4	6.10 (2.77–13.43)	<0.001	4.33 (2.60–7.22)	<0.001
Q5	4.46 (1.52–13.10)	0.007	3.28 (1.78–6.05)	<0.001
Panel B. Expanded sensitivity models including both focal variables and all nine contextual items (Q11–Q19) simultaneously				
Q3	3.46 (1.48–8.08)	0.004	1.31 (0.76–2.27)	0.327
Q4	5.13 (2.04–12.91)	<0.001	2.87 (1.63–5.07)	<0.001
Q5	3.41 (1.07–10.91)	0.038	2.71 (1.39–5.27)	0.003

Values are presented as adjusted odds ratios (aORs) with 95% confidence intervals (CIs) from logistic regression models. Panel A models were adjusted for age, sex, practice type, and specialty. Models with strict misuse-and-abuse classification as the focal variable included 300 physicians, whereas models with a restrictive view as the focal variable included 297 physicians after exclusion of the three respondents who selected "Unsure" for Q2. Panel B models included 297 physicians and contained both focal variables, age, sex, practice type, specialty, and all nine contextual items (Q11–Q19). Strict misuse-and-abuse classification was defined as Q1 response option 1 versus response options 2–4, and a restrictive view as Q2 response option 1 versus response options 2 and 3. All *p*-values are two-sided and nominal, with no false discovery rate adjustment. The 95% CIs were nominal and were not adjusted for multiple comparisons. Q3, prior recommendation of dose reduction or discontinuation; Q4, belief that dose reduction would be helpful; Q5, future intention to attempt dose reduction.

Supplementary Table S3. Adjusted predicted probabilities of physician-reported dose reduction-related responses across the four combined response patterns.

Combined response pattern	*n*	Prior recommendation of dose reduction or discontinuation (Q3)	Belief that dose reduction would be helpful (Q4)	Future intention to attempt dose reduction (Q5)
Strict–restrictive	27	76.7 (61.2–87.3)	85.9 (72.4–93.4)	92.7 (80.8–97.4)
Strict–non-restrictive	12	70.3 (51.9–83.8)	63.2 (42.4–80.1)	81.8 (60.5–92.9)
Non-strict–restrictive	80	52.8 (42.5–62.9)	57.9 (47.3–67.8)	81.4 (72.1–88.2)
Non-strict–non-restrictive	178	44.7 (37.8–51.8)	28.0 (22.0–34.9)	61.3 (54.2–68.0)

Values are adjusted predicted probabilities (%) with 95% confidence intervals (CIs), obtained from the primary mutually adjusted logistic regression models by marginal standardization; CIs were derived using the delta method. Each model included strict misuse-and-abuse classification, a restrictive view, age, sex, practice type, and specialty. No interaction term between strict misuse-and-abuse classification and a restrictive view was included. For each combined response pattern, the two focal variables were set to the corresponding combination, and predictions were averaged over the observed covariate distribution. The *n* column shows the observed number of physicians in each combined response pattern; predicted probabilities were estimated from the full analytic sample rather than calculated as subgroup-specific observed proportions. The first descriptor of each pattern refers to misuse-and-abuse classification, and the second refers to the view on how broadly medical narcotic use should be permitted. Analyses included the 297 physicians who selected a Q2 response other than “Unsure.” No formal between-pattern comparisons were performed.

Supplementary Table S4. Exploratory associations of individual contextual items (Q11–Q19) with strict misuse-and-abuse classification and a restrictive view.

Panel A. Outcome: strict misuse-and-abuse classification

Item group	Question	Outcome among "Yes" respondents, *n/N* (%)	Outcome among "No" respondents, *n/N* (%)	aOR (95% CI)	FDR-adjusted *p*-value
Narcotics knowledge and awareness	Q11	11/176 (6.2)	31/124 (25.0)	0.19 (0.09–0.41)	<0.001
	Q12	37/272 (13.6)	5/28 (17.9)	0.67 (0.23–1.93)	0.672
	Q13	29/240 (12.1)	13/60 (21.7)	0.47 (0.22–0.99)	0.139
Regulatory and control-system-related awareness	Q14	12/112 (10.7)	30/188 (16.0)	0.60 (0.29–1.25)	0.390
	Q15	36/243 (14.8)	6/57 (10.5)	1.52 (0.60–3.88)	0.672
	Q16	13/103 (12.6)	29/197 (14.7)	0.81 (0.39–1.66)	0.672
	Q17	17/120 (14.2)	25/180 (13.9)	0.96 (0.48–1.90)	0.951
Perceived patient misuse and related use patterns	Q18	3/30 (10.0)	39/270 (14.4)	0.68 (0.19–2.40)	0.672
	Q19	5/52 (9.6)	37/248 (14.9)	0.64 (0.24–1.74)	0.672

Panel B. Outcome: restrictive view

Item group	Question	Outcome among "Yes" respondents, *n/N* (%)	Outcome among "No" respondents, *n/N* (%)	aOR (95% CI)	FDR-adjusted *p*-value
Narcotics knowledge and awareness	Q11	49/175 (28.0)	58/122 (47.5)	0.42 (0.26–0.69)	0.005
	Q12	96/269 (35.7)	11/28 (39.3)	0.83 (0.37–1.86)	0.726
	Q13	83/237 (35.0)	24/60 (40.0)	0.79 (0.44–1.43)	0.672
Regulatory and control-system-related awareness	Q14	40/110 (36.4)	67/187 (35.8)	0.99 (0.60–1.63)	0.972
	Q15	98/241 (40.7)	9/56 (16.1)	3.66 (1.69–7.92)	0.006
	Q16	45/101 (44.6)	62/196 (31.6)	1.74 (1.05–2.89)	0.117
	Q17	49/119 (41.2)	58/178 (32.6)	1.48 (0.90–2.43)	0.311
Perceived patient misuse and related use patterns	Q18	9/30 (30.0)	98/267 (36.7)	0.78 (0.34–1.79)	0.672
	Q19	11/52 (21.2)	96/245 (39.2)	0.42 (0.20–0.87)	0.086

Values are presented as *n/N* (%) or adjusted odds ratios (aORs) with 95% confidence intervals (CIs). In the *n/N* (%) columns, *n* is the number of respondents with the panel outcome, and *N* is the number of respondents who answered "Yes" or "No" to the contextual item. Each aOR compares "Yes" with "No" in a separate logistic regression model adjusted for age, sex, practice type, and specialty. Panel A included all 300 physicians; Panel B included 297 physicians after exclusion of the three respondents who selected "Unsure" for Q2. Strict misuse-and-abuse classification was defined as Q1 response option 1 versus response options 2–4, and a restrictive view was defined as Q2 response option 1 versus response options 2 and 3. The contextual groupings were used for analytic organization only; internal-consistency estimates did not support treating them as coherent scales [15]. The marginal distributions of responses to Q11–Q19 were reported previously [15], whereas the adjusted associations shown here are new to the present analysis. FDR-adjusted *p*-values were calculated using the Benjamini–Hochberg procedure across all 18 item-level associations shown in Panels A and B; the 95% CIs were nominal and were not adjusted for multiple comparisons. Question numbers correspond to the full item wording and response options provided in Supplementary Appendix S3. FDR, false discovery rate.

Supplementary Figure S1. Exploratory associations of system-context items with physician-reported dose reduction-related responses. Points represent adjusted odds ratios (aORs), and horizontal lines represent 95% confidence intervals (CIs) for awareness of the NIMS Data Service (Q16) and willingness to try the NIMS Data Service (Q17). Separate logistic regression models were fitted for prior recommendation of dose reduction or discontinuation (Q3), belief that dose reduction would be helpful (Q4), and future intention to attempt dose reduction (Q5). Each model included Q16 and Q17 simultaneously and was adjusted for strict misuse-and-abuse classification, a restrictive view, age, sex, practice type, and specialty. Only estimates for Q16 and Q17 are displayed; estimates for the adjustment variables are not shown. All models included the 297 physicians who selected a Q2 response other than "Unsure." Adjusted odds ratios are displayed on a logarithmic scale, and the vertical dashed line indicates an aOR of 1.0. All hypothesis tests were two-sided and based on nominal *p*-values, without false discovery rate adjustment. CI, confidence interval; NIMS, Narcotics Information Management System.

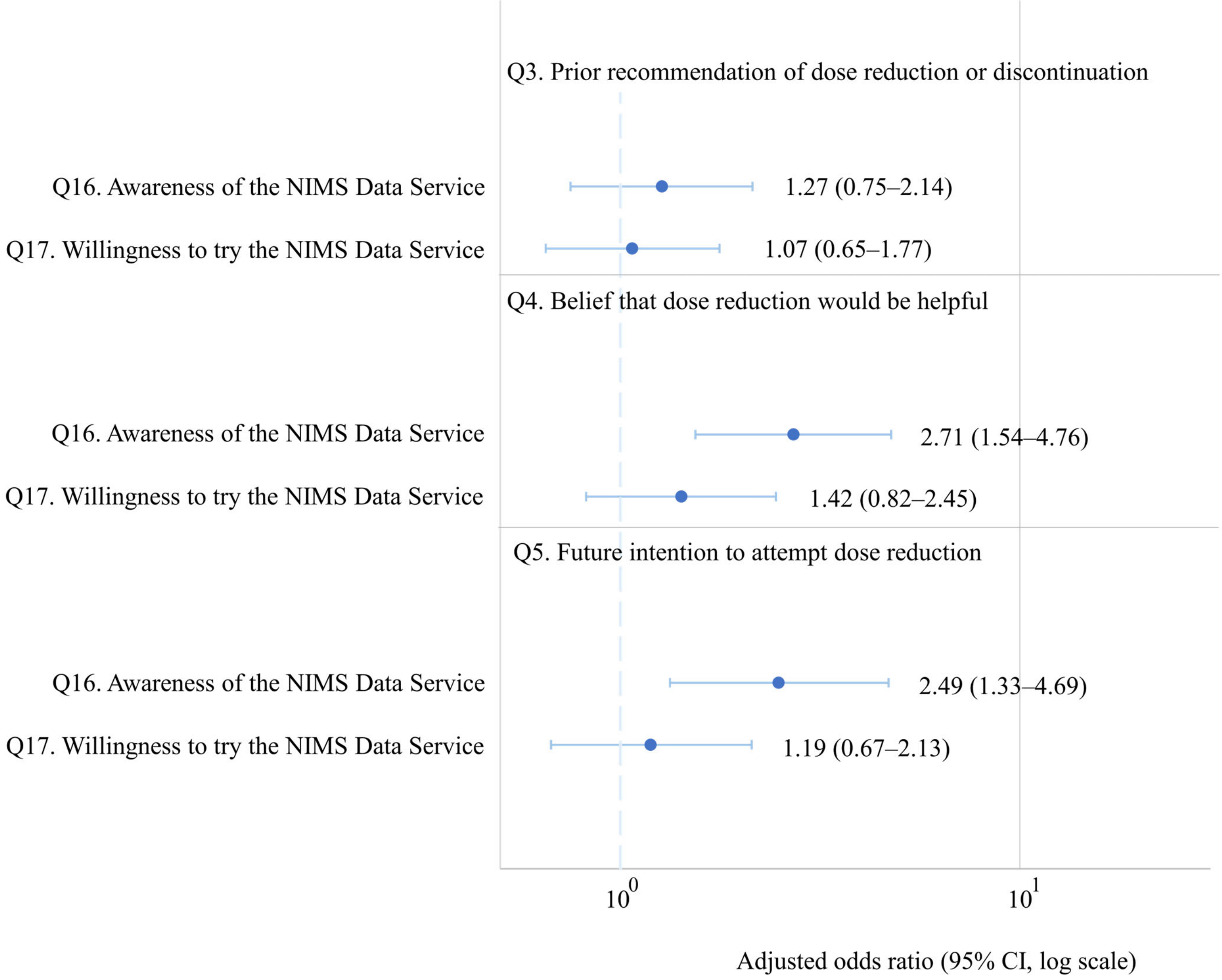

Supplementary Appendix S1. STROBE checklist

STROBE Statement–Checklist of Items That Should Be Included in Reports of Cross-Sectional Studies

Page numbers refer to the submitted manuscript. Supplementary tables, figures, and appendices are identified by their labels.

	Item No	Recommendation	page
Title and abstract	1	(*a*) Indicate the study's design with a commonly used term in the title or the abstract	p.1
		(*b*) Provide in the abstract an informative and balanced summary of what was done and what was found	p.1
Introduction			
Background/rationale	2	Explain the scientific background and rationale for the investigation being reported	p.2
Objectives	3	State specific objectives, including any prespecified hypotheses	p.2
Methods			
Study design	4	Present key elements of study design early in the paper	pp.2-3
Setting	5	Describe the setting, locations, and relevant dates, including periods of recruitment, exposure, follow-up, and data collection	pp.2-3
Participants	6	(*a*) Give the eligibility criteria, and the sources and methods of selection of participants	p.3
Variables	7	Clearly define all outcomes, exposures, predictors, potential confounders, and effect modifiers. Give diagnostic criteria, if applicable	pp.3-4
Data sources/ measurement	8*	For each variable of interest, give sources of data and details of methods of assessment (measurement). Describe comparability of assessment methods if there is more than one group	pp.3-4, Supplementary Appendix S3
Bias	9	Describe any efforts to address potential sources of bias	p.3, pp.10-11
Study size	10	Explain how the study size was arrived at	p.3
Quantitative variables	11	Explain how quantitative variables were handled in the analyses. If applicable, describe which groupings were chosen and why	p.4
Statistical methods	12	(*a*) Describe all statistical methods, including those used to control for confounding	pp.4-5
		(*b*) Describe any methods used to examine subgroups and interactions	N/A. no subgroup or interaction analyses were performed.
		(*c*) Explain how missing data were addressed	p.4

		(*d*) If applicable, describe analytical methods taking account of sampling strategy	N/A. no complex sampling design, weighting, or clustering adjustment was used.
		(*e*) Describe any sensitivity analyses	p.5, pp.7-8, Supplementary Table S2
Results			
Participants	13*	(a) Report numbers of individuals at each stage of study—eg numbers potentially eligible, examined for eligibility, confirmed eligible, included in the study, completing follow-up, and analysed	p.3
		(b) Give reasons for non-participation at each stage	p.3, p.10
		(c) Consider use of a flow diagram	No flow diagram. Participant flow is reported in text on p.3.
Descriptive data	14*	(a) Give characteristics of study participants (eg demographic, clinical, social) and information on exposures and potential confounders	pp.5-6
		(b) Indicate number of participants with missing data for each variable of interest	p.4
Outcome data	15*	Report numbers of outcome events or summary measures	pp.6-8
Main results	16	(*a*) Give unadjusted estimates and, if applicable, confounder-adjusted estimates and their precision (eg, 95% confidence interval). Make clear which confounders were adjusted for and why they were included	pp.7-8, Supplementary Table S1-S2
		(*b*) Report category boundaries when continuous variables were categorized	N/A. no continuous variable was categorized for the regression analyses.
		(*c*) If relevant, consider translating estimates of relative risk into absolute risk for a meaningful time period	Not directly applicable to this cross-sectional survey; adjusted predicted probabilities are reported on pp. 7-8 and Supplementary Table S3.
Other analyses	17	Report other analyses done—eg analyses of subgroups and interactions, and sensitivity analyses	pp.7-8, Supplementary Table S2-S4 and Figure S1
Discussion			
Key results	18	Summarise key results with reference to study objectives	pp.8-11

Limitations	19	Discuss limitations of the study, taking into account sources of potential bias or imprecision. Discuss both direction and magnitude of any potential bias	pp.10-11
Interpretation	20	Give a cautious overall interpretation of results considering objectives, limitations, multiplicity of analyses, results from similar studies, and other relevant evidence	pp.8-11
Generalisability	21	Discuss the generalisability (external validity) of the study results	pp.10-11
Other information			
Funding	22	Give the source of funding and the role of the funders for the present study and, if applicable, for the original study on which the present article is based	p.11

*Give information separately for exposed and unexposed groups, where applicable.

Supplementary Appendix S2. CHERRIES checklist

CHERRIES–Checklist for Reporting Results of Internet E-Surveys

Page numbers refer to the submitted manuscript. Supplementary table, figures, and appendices are identified by their labels.

Checklist item	Reporting recommendation	Page Number
Design		
Describe survey design	Describe the target population, sampling frame, and whether the sample was open, convenience-based, or otherwise selected.	pp.2-3
IRB approval and informed consent process		
IRB approval	State whether institutional review board or ethics approval was obtained.	p.3, p.11
Informed consent	Describe the electronic consent process and the information given to participants, including study purpose, expected survey length, voluntariness, and data handling.	p.3, p.11
Data protection	Describe safeguards used to protect personal or identifiable information and prevent unauthorized access.	p.3
Development and pre-testing		
Development and testing	Explain how the questionnaire was developed and pretested, including usability or technical testing of the electronic survey when performed.	pp.3-4
Recruitment process and description of the sample having access to the questionnaire		
Open survey versus closed survey	State whether the survey was open to any visitor or restricted to a known invited sample.	p.3
Contact mode	Indicate whether initial contact with potential participants occurred online and describe the contact method.	p.3
Advertising the survey	Describe how and where the survey was announced or advertised and, when relevant, the wording or channel used.	p.3
Survey administration		
Web/E-mail	State the form of electronic survey and how responses were captured.	p.3
Context	Describe the website, mailing list, or other setting through which respondents accessed the survey and how that context may have shaped the sample.	p.3
Mandatory/voluntary	State whether participation was mandatory or voluntary.	p.3

Incentives	Report any monetary, prize-based, or nonmonetary incentives.	p.3
Time/Date	Report the period during which data were collected.	p.3
Randomization of items or questionnaires	State whether the order of items or questionnaire versions was randomized or alternated.	Not reported.
Adaptive questioning	Describe conditional display or skip logic used to tailor questions to earlier responses.	p.3
Number of Items	Report the number of questionnaire items shown per screen or page.	Supplementary Appendix S3
Number of screens (pages)	Report the number of screens or pages over which the questionnaire was distributed.	Not reported.
Completeness check	Describe whether completeness or internal-consistency checks were performed before or after submission and how missing responses were handled technically.	pp.4-5
Review step	State whether respondents could review and change answers before final submission.	Not reported.
Response rates		
Unique site visitor	When site-based rates are reported, define how unique visitors were identified.	p.3
View rate	Report the ratio of unique survey-page visitors to unique site visitors when applicable.	N/A.
Participation rate	Report the proportion of unique first-page or consent-page visitors who agreed to participate.	p.3
Completion rate	Report the proportion of participants who finished the survey among those who agreed to participate or began the questionnaire.	p.3
Preventing multiple entries from the same individual		
Cookies used	State whether cookies or browser identifiers were used to detect or prevent duplicate submissions.	Not reported.
IP check	State whether Internet Protocol addresses were used to identify or prevent duplicate entries.	Not reported.
Log file analysis	Describe any log-file analysis or other methods used to detect multiple entries.	Not reported.
Registration	For a closed survey, describe login, registration, or other respondent-specific controls used to prevent repeat participation.	p.3
Analysis		
Handling of incomplete questionnaires	State whether only complete questionnaires were analyzed and how early terminations or partially completed surveys were handled.	p.3

Questionnaires submitted with an atypical timestamp	Describe whether completion time was examined and whether unusually rapid or slow submissions were excluded.	Not reported.
Statistical correction	Describe weighting, propensity scores, or other methods used to correct for non-representativeness of the web-survey sample.	pp.4-5

Supplementary Appendix S3. Wording and response options of survey items used in the present study

Item	Wording	Response options
Q1. Misuse-and-abuse classification of off-label use	Which of the following statements most closely matches your definition of misuse and abuse involving medical narcotics?	(1) All use of medical narcotics beyond the label approved by the Ministry of Food and Drug Safety (MFDS) should be regarded as misuse and abuse<br>(2) Use of medical narcotics beyond the MFDS-approved label should not be regarded as misuse and abuse if it is recognized in clinical guidelines<br>(3) Use of medical narcotics beyond the MFDS-approved label should not be regarded as misuse and abuse if it is recognized in textbooks or other standard references<br>(4) Use of medical narcotics beyond the MFDS-approved label should not be regarded as misuse and abuse if prescribed by a physician
Q2. View on how broadly medical narcotic use should be permitted	Given societal concerns about misuse and abuse, which statement best reflects your view on how broadly medical narcotic use should be permitted?	(1) Medical narcotic use, including off-label use, should be restricted because of serious societal concerns about misuse and abuse<br>(2) Medical narcotic use, including off-label use, should be permitted at the physician's discretion<br>(3) Medical narcotic use, including off-label use, should be permitted when there is a clear medical need<br>(4) Unsure
Q3. Prior recommendation of dose reduction or discontinuation	Have you previously recommended dose reduction or discontinuation of medical narcotics?	Yes/No

Q4. Belief that dose reduction would be helpful	Do you believe that dose reduction of medical narcotics would be helpful?	Yes/No
Q5. Future intention to attempt dose reduction	Do you intend to attempt to reduce the dose of medical narcotics in the future?	Yes/No
Q6. Age	What is your age?	Free-text numeric responses
Q7. Years in practice	How many years of clinical experience do you have?	Free-text numeric responses
Q8. Sex	What is your sex?	Male/Female
Q9. Practice type	What is your practice type?	Private practitioner / University hospital physician / Employed physician
Q10. Specialty	What is your medical specialty?	Psychiatry / Neurology / Anesthesiology and pain medicine
Q11. Distinguishing medical narcotics from illicit drugs	Can you distinguish medical narcotics from illicit drugs?	Yes/No
Q12. Awareness of medical narcotics classification	Are you aware that medical narcotics are subject to legal classification and regulation?	Yes/No
Q13. Awareness of NIMS reporting	Are you aware that prescribing of medical narcotics must be reported to the Narcotics Information Management System (NIMS)?	Yes/No
Q14. Awareness of the narcotics prescription status inquiry system	Are you aware of the narcotics prescription status inquiry system?	Yes/No

Q15. Awareness of physicians' right to decline a prescription request	Are you aware that physicians may decline a request for a medical narcotic prescription when the prescription would be clinically inappropriate?	Yes/No
Q16. Awareness of the NIMS Data Service	Are you aware of the NIMS Data Service, which provides information on a patient's medical narcotic prescription history?	Yes/No
Q17. Willingness to try the NIMS Data Service	Would you be willing to try using the NIMS Data Service in clinical practice?	Yes/No
Q18. Perceived patient misuse and abuse of prescribed medical narcotics	Have you suspected misuse and abuse of prescribed medical narcotics among your patients?	Yes/No
Q19. Awareness of dose increases since treatment initiation	Have you recognized cases in which a patient's prescribed dose of medical narcotics increased after treatment initiation?	Yes/No

The original survey was administered in Korean. The English wording provided here was lightly edited for readability while preserving the substantive meaning of the original items. The original Korean questionnaire used a single expression encompassing both misuse and abuse; accordingly, "misuse and abuse" is used in this appendix as an umbrella term and does not denote separate legal, psychiatric, or diagnostic categories.
