# Supplementary material for: Physicians’ Judgments About Off-Label Medical Narcotic Use and Dose Reduction Responses: A South Korean Cross-Sectional Survey
Source: Medicina (Kaunas). 2026 Sep 10;62(9):1748. doi: 10.3390/medicina62091748 (PMC13608935; doi:10.3390/medicina62091748)
Supplement: Supplementary file 1 [file medicina-62-01748-s001.zip › Questionnaire Form_ENG.pdf]

Survey of Patients' and Physicians' Perceptions of and Experiences with Medical Narcotics
(Physician Questionnaire)

**Demographic Information**			
Sex		Age	

Research Participant Information Sheet and Consent Form

Participant Information Sheet	
Study Title	Survey of Patients' and Physicians' Perceptions of and Experiences with Medical Narcotics
Principal Investigator	Jung Eun Kim

1. Study Overview and Participant Information

This survey study aims to examine physicians' perceptions of and experiences with the use and management of medical narcotics, including opioid analgesics and controlled psychotropic medications.

The principal investigator is Jung Eun Kim of Hallym University Kangnam Sacred Heart Hospital. For questions about the study, please contact the research team at 02-1577-5587.

Only individuals who voluntarily agree to participate may take part in this study. Before deciding whether to participate, it is important that you fully understand the purpose of the study, what participation will involve, what information will be collected and how it will be used, and any potential benefits, risks, or discomforts associated with participation. Please take sufficient time to read the following information carefully. Should you have any questions about the study, please contact the research team.

2. Background and Purpose of the Study

Medical narcotics are medications used for various clinical needs, including the treatment of pain, anxiety, and sleep disorders, and should not be equated with illicit drugs. However, because their use may involve risks of dependence, misuse and abuse, and other forms of inappropriate use, they must be used and managed safely and appropriately while ensuring that patients have access to necessary treatment. In South Korea, records concerning the handling of medical narcotics are reported and managed through the Narcotics Information Management System in accordance with the Narcotics Control Act.

The purpose of this study is to examine physicians' perceptions of and experiences with the use, misuse and abuse of medical narcotics, the narcotics control framework, and the Narcotics Information Management System. The study also aims to investigate physicians' clinical judgments and experiences concerning off-label use, the permitted scope of medical narcotic use, and appropriate use. Through this research, we aim to provide foundational evidence for the safe and appropriate use and management of medical narcotics.

3. Study Participants

A total of 800 board-certified physicians in anesthesiology and pain medicine, neurology, and psychiatry across South Korea will be invited by email to participate in the survey. Only physicians with prior experience prescribing medical narcotics will be eligible to participate; physicians without such experience will be excluded.

4. Study Procedures

Data will be collected through an online questionnaire accessed via a survey link provided by email. The questionnaire includes items concerning perceptions of the use and management of medical narcotics, prescribing-related experiences, and general participant characteristics. It is expected to take approximately 5–10 minutes to complete.

5. Potential Benefits of Participation

You will not receive any financial compensation or direct benefit from participating in this study. However, the information collected through this study may provide foundational evidence for improving the safe use of medical narcotics and related management systems and policies in South Korea.

6. Potential Risks and Discomforts of Participation

Because this study is conducted through an online questionnaire, no physical risks or direct harm resulting from participation are anticipated. However, completing the questionnaire will require some of your time, and you may experience discomfort when answering certain questions or have concerns about the privacy and confidentiality of the information collected.

If you have any questions about potential risks or discomforts related to your participation, please contact the research team.

7. Voluntary Participation

Your decision whether to participate in this study is entirely voluntary. You may decline to participate, and you will not experience any penalty or disadvantage for choosing not to participate. Even after agreeing to participate, you may stop completing the questionnaire at any time before submitting it, without any penalty or disadvantage.

8. Study-Related Questions and Provision of New Information

You may contact the research team at any time if you have questions about any aspect of the study. If new information becomes available during the study that may affect your willingness to participate, the research team will inform you promptly.

9. Management of Unexpected Problems During Participation

Because this study is conducted through an online questionnaire, no physical injury resulting from participation is anticipated. However, if you experience any unexpected problem or discomfort related to your participation, please inform the research team promptly.

10. Protection of Personal Information and Confidentiality

Your name will not be collected as part of the survey responses. General information, such as your sex, age, medical specialty, and type of practice, as well as other information collected through the questionnaire, will be assigned a study identification number and managed in coded form.

Only authorized members of the research team will have access to the electronic data collected, which will be protected through appropriate security measures, including password protection. However, authorized representatives of the Institutional Review Board and the Human Research Protection Center may review the research data to verify that the study is being conducted appropriately and to provide oversight. Your personal information and confidentiality will remain protected during any such review.

Study-related data will be retained for three years after completion of the study and will then be destroyed. Electronic data will be permanently deleted so that they cannot be recovered. By agreeing to participate in the study, you consent to the collection, use, storage, and authorized review of your information as described above.

11. Discontinuation of Participation and Handling of Data

You may discontinue your participation in the survey at any time before submitting the completed questionnaire. You will not experience any penalty or disadvantage for discontinuing your participation.

If you wish to withdraw from the study after submitting the questionnaire, please inform a member of the research team or the principal investigator. However, because this study does not

collect participants' names and manages survey responses in coded form, it may be difficult to identify your individual responses after submission. In addition, data that have already been de-identified, combined with data from other participants, or analyzed may no longer be individually identifiable or removable.

12. Study-Related Inquiries and Contact Information

If you have any questions about this study or if any problems related to the study arise, please contact the researcher below.

Principal Investigator: Jung Eun Kim<br>Affiliation: Department of Anesthesiology and Pain Medicine, Hallym University Kangnam Sacred Heart Hospital<br>Address: Department of Anesthesiology and Pain Medicine, Hallym University Kangnam Sacred Heart Hospital, 1 Singil-ro, Yeongdeungpo-gu, Seoul<br>Telephone: Jung Eun Kim 02-1577-5587<br>Clinical Research Ethics Committee Telephone: 02-829-5527

**Research Participation Consent Form**	
Study Title	Survey of Patients' and Physicians' Perceptions of and Experiences with Medical Narcotics
Subject Type	Physician Questionnaire
Principal Investigator	Jung Eun Kim

1. I have read the participant information sheet above and fully understand the purpose of the study and the procedures involved in participation.
2. I understand the potential benefits, risks, and discomforts associated with participation, as well as the measures taken to protect personal information and confidentiality.
3. I understand that I may ask questions about the study and receive sufficient explanations as needed.
4. I understand that my decision to participate is entirely voluntary, and that I may decline to participate or discontinue the survey at any time before final submission without any penalty or disadvantage.
5. I voluntarily agree to participate in this study and consent to the collection and use of my survey responses for research purposes.

□ I have read and understood the information above and voluntarily agree to participate in this study and to allow my survey responses to be used for research purposes.

Eligibility Screening

Have you ever prescribed medical narcotics, including opioid analgesics or controlled psychotropic medications?

1) Yes
2) No → You are not eligible to participate in this survey.

1. Which of the following statements most closely reflects your view of misuse or abuse in relation to medical narcotics?

   1) Any use beyond the Ministry of Food and Drug Safety (MFDS)-approved label should be regarded as misuse or abuse.

   2) Use beyond the MFDS-approved label should not be regarded as misuse or abuse if it is widely accepted in textbooks or standard medical references.

   3) Use beyond the MFDS-approved label should not be regarded as misuse or abuse if it is prescribed by a physician.

   4) Use beyond the MFDS-approved label may be allowed if it is prescribed by a physician.

2. What is your view on expanding the permitted scope of medical narcotic use?

   1) Misuse and abuse of medical narcotics are serous societal problem; therefore, the use of medical narcotics should be restricted to a certain extent.

   2) Measures should be established to prevent societal misuse and abuse, but prescribing medical narcotics should be left to the discretion of the prescribing physician.

   3) Even if particular use is socially perceived as misuse or abuse, the use of medical narcotics should be permitted when there is a clear medical necessity.

   4) Unsure.

3. In the past year, have you recommended reducing or discontinuing a patient's medical narcotic medication?

   1) Yes.

   2) No.

4. Do you think that reducing a patient's medical narcotic medication would generally be helpful?

   1) Yes.

   2) No.

5. Do you plan to attempt to reduce a patient's medical narcotic medication in your future practice?

   1) Yes.

   2) No.

6. What is your age?

_________ years

7. How many years of clinical experience do you have?

_________ years

8. What is your sex?

1) Male

2) Female

9. What is your current practice type?

1) Private practitioner

2) University hospital physician

3) Employed physician

10. What is your medical specialty?

1) Psychiatry

2) Neurology

3) Anesthesiology and Pain Medicine

11. Can you distinguish medical narcotics from illicit drugs?

1) Yes.

2) No.

12. Are you aware that medications prescribed to patients may classified as medical narcotics?

1) Yes.

2) No.

13. Are you aware that prescriptions for medical narcotics are reported to the Narcotics Information Management System (NIMS)?

1) Yes.

2) No.

14. Are you aware that physicians can access information on a patient's medical narcotic prescription history?

1) Yes.

2) No.

15. Are you aware that physicians may decline a request for a medical narcotic prescription when misuse or abuse is suspected?

1) Yes.

2) No.

16. Are you aware of the NIMS Data Service, which provides information on a patient's medical narcotic prescription history?

1) Yes.

2) No.

17. Would you be willing to use the NIMS Data Service in clinical practice?

1) Yes.

2) No.

18. Have you ever suspected misuse or abuse of prescribed medical narcotics among your patients?
    1) Yes.
    2) No.

19. Have you encountered patients whose prescribed dose of medical narcotics increased after treatment initiation?
    1) Yes.
    2) No.

Thank you.
