# Supplementary material for: Physicians’ Judgments About Off-Label Medical Narcotic Use and Dose Reduction Responses: A South Korean Cross-Sectional Survey
Source: Medicina (Kaunas). 2026 Sep 10;62(9):1748. doi: 10.3390/medicina62091748 (PMC13608935; doi:10.3390/medicina62091748)
Supplement: Supplementary file 1 [file medicina-62-01748-s001.zip › Questionnaire Form_KOR.pdf]

의료용 마약류에 대한 환자와 의사의 인식 및 관련 경험 조사

(의사용 설문지)

인적사항			
나이		성별	

연구대상자 설명문 및 동의서

연구대상자 설명문	
연구과제명	의료용 마약류에 대한 환자와 의사의 인식 및 관련 경험 조사
연구책임자	김정은

1. 연구 개요 및 참여 안내

본 연구는 의료용 마약류(마약성 진통제 및 향정신성의약품)의 사용과 관리에 대한 의사의 인식 및 관련 경험을 조사하기 위한 설문연구입니다.

본 연구의 연구책임자는 한림대학교 강남성심병원 김정은이며, 연구에 관한 문의사항은 전화번호 02-1577-5587로 연락하여 주시기 바랍니다.

본 연구에는 자발적으로 참여 의사를 밝힌 분만 참여할 수 있습니다. 연구 참여 여부를 결정하기에 앞서, 본 연구의 목적과 참여 과정, 수집되는 정보와 그 이용 방법, 그리고 연구 참여로 예상되는 이점과 위험 또는 불편에 대해 충분히 이해하는 것이 중요합니다. 다음 내용을 시간을 두고 주의 깊게 읽어 주시기 바랍니다. 연구와 관련하여 궁금한 사항이 있으면 연구진에게 문의하여 주십시오.

2. 연구의 배경과 목적

의료용 마약류는 통증, 불안, 수면장애 등 다양한 임상적 필요에 따라 사용되는 의약품으로, 불법약물과 동일한 개념은 아닙니다. 그러나 의료용 마약류에는 의존, 오남용 및 부적절한 사용의 가능성이 있으므로, 환자에게 필요한 치료를 보장하면서도 안전하

고 적절하게 사용하고 관리할 필요가 있습니다. 국내에서는 “마약류 관리에 관한 법률”에 따라 의료용 마약류의 취급 내역을 마약류통합관리시스템을 통해 보고하고 관리하고 있습니다.

본 연구는 의료용 마약류의 사용과 오남용, 마약류 관리제도 및 마약류통합관리시스템에 대한 의사의 인식과 관련 경험을 파악하는 것을 목적으로 합니다. 또한 의료용 마약류의 허가사항 외 사용과 허용 범위, 그리고 적정 사용을 둘러싼 임상적 판단과 경험을 조사하고자 합니다. 이를 통해 의료용 마약류의 안전하고 적절한 사용과 관리에 필요한 기초자료를 마련하고자 합니다.

3. 연구 참여 대상

본 연구는 전국의 마취통증의학과, 신경과 및 정신건강의학과 전문의 800명에게 이메일로 설문 참여를 요청할 예정입니다. 의료용 마약류를 처방한 경험이 있는 의사만 연구에 참여할 수 있으며, 의료용 마약류 처방 경험이 없는 의사는 참여 대상에서 제외됩니다.

4. 연구 방법

본 연구에서는 이메일로 전달된 설문 링크를 통해 온라인으로 자료를 수집합니다. 설문에는 의료용 마약류의 사용과 관리에 대한 인식, 처방 관련 경험 및 일반적 특성에 관한 문항이 포함됩니다. 설문 작성에는 약 5–10분이 소요됩니다.

5. 연구 참여에 따른 이익

귀하가 본 연구에 참여함으로써 얻는 금전적 보상이나 직접적인 이익은 없습니다. 다만, 본 연구를 통해 수집된 정보는 향후 국내 의료용 마약류의 안전한 사용과 관

리제도 및 관련 정책을 개선하기 위한 기초자료로 활용될 수 있습니다.

6. 연구 참여에 따른 위험 및 불편

본 연구는 온라인 설문조사로 진행되므로 연구 참여로 인한 신체적 위험이나 직접적인 피해는 없을 것으로 예상됩니다. 다만 설문지 작성에 시간이 소요되며, 일부 질문에 답하는 과정에서 불편함을 느끼거나 수집된 정보의 개인정보 보호 및 비밀 유지에 대해 우려할 수 있습니다.

연구 참여와 관련하여 발생할 수 있는 위험이나 불편에 대해 궁금한 사항이 있으면 연구진에게 문의하여 주십시오.

7. 연구 참여의 자발성

본 연구의 참여 여부는 귀하의 자발적인 의사에 따라 결정됩니다. 귀하는 연구 참여를 거절할 수 있으며, 참여하지 않더라도 어떠한 불이익도 받지 않습니다. 연구 참여에 동의한 이후에도 설문 제출 전까지 언제든지 응답을 중단할 수 있으며, 이로 인한 어떠한 불이익도 발생하지 않습니다.

8. 연구 관련 문의 및 새로운 정보의 제공

연구의 세부 사항에 대해 궁금한 점이 있는 경우 언제든지 연구진에게 문의할 수 있습니다. 연구 기간 중 귀하의 연구 참여 여부에 영향을 줄 수 있는 새로운 정보가 확인되면, 연구진은 해당 정보를 귀하에게 신속하게 알려 드릴 것입니다.

9. 연구 참여 중 예기치 않은 문제 발생 조치

본 연구는 온라인 설문조사로 진행되므로 연구 참여로 인한 신체적 손상은 예상되지

않습니다. 다만 연구 참여와 관련하여 예기치 않은 문제나 불편이 발생한 경우에는 연구진에게 즉시 알려 주시기 바랍니다.

10. 개인정보 보호 및 비밀 보장

본 연구의 설문 응답자료에는 귀하의 성명을 수집하지 않습니다. 성별, 나이, 진료과, 근무 형태 등의 일반적 정보와 설문을 통해 수집된 정보에는 연구용 번호를 부여하여 관리합니다.

수집된 전자자료에는 접근 권한이 부여된 연구진만 접근할 수 있으며, 비밀번호 설정 등 적절한 보안조치를 통해 보호합니다. 다만 연구가 적절하게 수행되고 있는지 확인하고 관리 감독하기 위하여 기관생명윤리위원회 및 피험자보호센터의 권한을 부여받은 관계자가 연구자료를 검토할 수 있습니다. 이 경우에도 귀하의 개인정보와 비밀은 보호됩니다.

연구 관련 자료는 연구 종료 후 3년간 보관한 뒤 폐기하며, 전자자료는 복구할 수 없도록 영구적으로 삭제합니다. 귀하는 연구 참여에 동의함으로써 위와 같은 정보의 수집, 이용, 보관 및 권한 있는 관계자의 검토에 동의하게 됩니다.

11. 연구 참여 중단 및 자료 처리

귀하는 설문을 최종 제출하기 전까지 언제든지 설문 참여를 중단할 수 있습니다. 참여를 중단하더라도 귀하에게 어떠한 불이익도 발생하지 않습니다.

설문을 제출한 이후 연구 참여 철회를 원하는 경우 담당 연구원 또는 연구책임자에게 알려주시기 바랍니다. 다만 본 연구에서는 성명을 수집하지 않고 응답자료를 코드화하여 관리하므로, 설문 제출 후에는 귀하의 응답을 개별적으로 확인하기 어려울 수 있습니다. 또한 이미 비식별화되었거나 다른 참여자의 자료와 통합 또는 분석

된 자료는 개별적으로 확인하여 삭제하기 어려울 수 있습니다.

12. 연구 관련 문의 및 연락처

본 연구에 관하여 궁금한 점이 있거나 연구 참여와 관련된 문제가 발생한 경우에는 아래의 연구책임자에게 문의하여 주시기 바랍니다.

연구책임자: 김정은

소속: 한림대학교 강남성심병원 마취통증의학과

주소: 서울특별시 영등포구 신길로 1, 한림대학교 강남성심병원 마취통증의학과

전화번호: 김정은 02-1577-5587

임상시험연구윤리위원회 전화번호 02-829-5527

연구 참여 동의서	
연구과제명	의료용 마약류에 대한 환자와 의사의 인식 및 관련 경험 조사
설문 유형	의사용 설문지
연구책임자	김정은

1. 본인은 위의 연구대상자 설명문을 읽었으며, 본 연구의 목적과 참여 방법에 대한 설명을 충분히 이해하였습니다.
2. 본인은 연구 참여에 따른 잠재적 이익, 위험 및 불편과 개인정보 보호 및 비밀 보장에 관한 내용을 이해하였습니다.
3. 본인은 연구와 관련하여 궁금한 사항을 문의할 수 있으며, 필요한 경우 충분한 설명을 받을 수 있음을 이해하였습니다.
4. 본인은 연구 참여 여부가 본인의 자발적인 의사에 따라 결정되며, 연구 참여를 거절하거나 설문을 최종 제출하기 전까지 언제든지 참여를 중단하여도 어떠한 불이익도 받지 않음을 이해하였습니다.
5. 본인은 본 연구에 자발적으로 참여하며, 본인의 설문 응답이 연구 목적으로 수집, 이용되는 것에 동의합니다.

□ 본인은 위의 설명을 읽고 이해하였으며, 본 연구에 자발적으로 참여하고 본 설문의 응답이 연구 목적으로 사용되는 것에 동의합니다.

설문 참여 자격 확인

귀하는 의료용 마약류(마약성 진통제 또는 향정신성의약품)를 처방한 경험이 있습니까?

1) 예

2) 아니오 → 설문 종료

1. 다음 중 의료용 마약류의 오남용에 대한 선생님의 생각과 가장 가까운 것은 무엇입니까?

1) 식품의약품안전처(식약처)의 허가사항을 벗어난 모든 의료용 마약류 사용은 오남용으로 보아야 한다.

2) 식약처의 허가사항을 벗어난 사용이라도 교과서 또는 표준적인 의학 참고문헌에서 널리 인정되는 경우에는 오남용으로 보아서는 안된다.

3) 식약처의 허가 사항을 벗어난 사용이라도 의사가 처방한 경우에는 오남용으로 보아서는 안된다.

4) 식약처 허가사항을 벗어난 사용이라도 의사가 처방한 경우에는 허용될 수 있다.

2. 선생님은 의료용 마약류의 허용 범위를 확대하는 것에 대해 어떻게 생각하십니까?

1) 의료용 마약류의 오남용은 심각한 사회적 문제이므로, 의료용 마약류의 사용은 일정 수준 제한되어야 한다.

2) 사회적 오남용을 방지하기 위한 대책은 마련되어야 하지만, 의료용 마약류의 처방은 처방 의사의 판단에 맡겨야 한다.

3) 사회적으로 오남용으로 인식되는 사용이라 하더라도, 명확한 의학적 필요성이 있는 경우에는 의료용 마약류의 사용을 허용해야 한다.

4) 모르겠다.

3. 최근 1년간, 환자에게 마약류 의약품의 감량 또는 중단을 권고한 적이 있습니까?

1) 예

2) 아니오

4. 환자의 마약류 의약품을 감량하는 것이 전반적으로 도움이 된다고 생각하십니까?

1) 예

2) 아니오

5. 향후 진료에서 환자의 마약류 의약품을 감량하려고 시도할 계획이 있으십니까?

1) 예

2) 아니오

6. 귀하의 연령은 몇 세 입니까?

만 _______________ 세

7. 귀하의 임상 진료 경력은 몇 년입니까?

________ 년

8. 귀하의 성별은 무엇입니까?

1) 남성
2) 여성

9. 귀하의 현재 근무 형태는 무엇입니까?

1) 개원의
2) 대학병원 근무의
3) 봉직의

10. 귀하의 전문 진료과는 무엇입니까?

1) 정신건강의학과
2) 신경과
3) 마취통증의학과

11. 선생님은 의료용 마약류와 불법 마약의 차이를 구분할 수 있습니까?

1) 예

2) 아니오

12. 선생님은 환자에게 처방하는 의약품이 의료용 마약류로 분류될 수 있다는 사실을 알고 계십니까?

1) 예

2) 아니오

13. 의료용 마약류의 처방 정보가 마약류통합관리시스템(NIMS)에 보고된다는 사실을 알고 계십니까?

1) 예

2) 아니오

14. 선생님은 의사가 환자의 마약류 의약품 처방 현황을 조회할 수 있는 제도가 있다는 사실을 알고 계십니까?

1) 예

2) 아니오

15. 환자의 의료용 마약류 오남용이 의심되는 경우, 의사가 처방을 거부할 수 있다는 사실을 알고 계십니까?

1) 예

2) 아니오

16. 환자의 의료용 마약류 처방 이력을 확인할 수 있는 마약류 의료쇼핑 방지 정보망 서비스에 대해 알고 계십니까?

1) 예

2) 아니오

17. 마약류 의료쇼핑 방지정보망 서비스를 진료에 이용해 볼 의향이 있습니까?

1) 예

2) 아니오

18. 진료 중인 환자가 처방받은 의료용 마약류를 오남용한다고 의심한 적이 있습니까?

1) 예

2) 아니오

19. 의료용 마약류를 처방받은 환자에서 치료 시작 후 처방 용량이 증가한 경우를 경험한 적이 있습니까?

1) 예

2) 아니오

감사합니다.
